# Diffusion‐weighted imaging‐documented bilateral small embolic stroke involving multiple vascular territories may indicate occult cancer: A retrospective case series and a brief review of the literature

**DOI:** 10.1002/agm2.12105

**Published:** 2020-03-25

**Authors:** Xiaosa Chi, Renliang Zhao, Haitao Pei, Ang Xing, Song Hu, Jingjiao Chen, Yongjun Mao, Xueping Zheng

**Affiliations:** ^1^ Department of Geriatrics Affiliated Hospital of Qingdao University Qingdao China; ^2^ Department of Neurology Affiliated Hospital of Qingdao University Qingdao China

**Keywords:** cancer, diffusion‐weighted imaging, embolic stroke, first manifestation

## Abstract

Diffusion‐weighted imaging (DWI) MRI is very sensitive for detecting small embolic brain infarctions. Stroke as the first manifestation of cancer is extremely rare. We performed a retrospective study to identify the clinical and DWI features of patients with acute ischemic stroke as the first manifestation of occult cancer. A total of five patients in our hospital from January 2017 to May 2019 were analyzed. We also reviewed the literature and seven case series (16 patients) were included. Most of these patients were aged in their sixties and lung cancer was the most common type of occult cancer. Patients showed various presentations of ischemic stroke. All of the patients showed small multiple lesions on DWI that involved mostly the anterior or both anterior and posterior territories. The lesions were mostly in both the supratentorium and infratentorium, with the mechanisms of embolic and watershed infarcts. These features were useful for identifying the causes of embolic stroke. Therefore, patients with small bilateral embolic stroke, especially those involved in multiple vascular territories, should be examined for concealed malignancy.

## 1 INTRODUCTION

Diffusion‐weighted imaging (DWI) MRI is a sensitive and indispensable tool for evaluating embolic brain infarctions, especially small single or multiple infarcts in the cortex or subcortex.^1^ Cardiogenic, paradoxical, and aortogenic emboli are common sources of embolic stroke. These emboli can be detected with transesophageal echocardiography (TEE). However, the embolic sources of 16% of patients with embolic stroke could not be identified.^2^, ^3^ The occurrence of embolic stroke is common in cancer patients during their clinical course. The rate of stroke in patients with cancer is twice that of the general population, as the survival rates of cancer patients have increased in recent decades.^4^ Hypercoagulability, tumor compression of blood, non‐bacterial thrombotic endocarditis, and therapy‐related effects are the main mechanisms of stroke in cancer.^5^ However, stroke encountered as the first manifestation of cancer is very rare. Little research has been done on the DWI presentations of cancer‐related stroke. Here, we report five cancer patients with ischemic stroke as the first presentation. We also briefly review the literature on this topic.

## 2 PATIENTS AND METHODS

This study included all patients who had presented with acute brain infarction and had been first diagnosed with cancer at the time of stroke presentation at the Department of Neurology of the Affiliated Hospital of the Medical School of Qingdao University from January 2017 to May 2019. Symptom duration was not considered for patient inclusion as long as an acute infarct was identified in DWI.^6^ Patients with known cancer who were admitted because of a new stroke were excluded. Patients whose cancers had been diagnosed 1 month after the presentation of acute ischemic stroke were also excluded. A total of five patients were analyzed. None of them had cancer history. All patients underwent brain DWI. Magnetic resonance (MR) angiography, computed tomography (CT) angiography, or transcranial Doppler ultrasonography was performed to evaluate the vessels. DWI findings were evaluated by a neuroradiologist blinded to the clinical symptoms and TEE findings. Lesions were considered small when the largest axial diameter was <15 mm, and large if ≥15 mm. Lesions were considered multiple if they were noncontiguous on contiguous slices.

We also systematically searched the studies reporting on embolic stroke as the first manifestation of cancer in PubMed (until May 5, 2019). The following search terms were used: (“stroke,” “cancer,” “first manifestation”) or (“stroke,” “malignancy,” “first manifestation”) or (“stroke,” “cancer”, “initial manifestation”) or (“stroke,” “malignancy,” “initial manifestation”). Studies reporting stroke as the first presentation of cancer and pictures of DWI were included for further evaluation. Each patient’s DWI picture was reviewed by a neuroradiologist.

## 3 CASE REPORT

### 3.1 Case 1

A 75‐year‐old man was admitted to our hospital because of dysarthria, which had begun 4 days prior to admission. He had no history of hypertension, diabetes, or heart disease, no familial history of cardiovascular disease, and no use of preventive medication for stroke.

On admission, he was oriented and had no lymphadenopathy, no abnormal auscultation respiratory sound, and no palpable masses in his abdomen. His vital signs were normal. There was no abnormality on the electrocardiogram (ECG). His mental status was alert, and other aspects of the neurologic examination were normal, except for the presence of dysarthria, right central glossary palsy, and bilateral positive Babinski signs.

Laboratory tests showed hyperlipidemia, but the results of other tests were all normal, including tests of serum electrolytes, renal and hepatic function, blood sugar, coagulation profile (including activated partial thromboplastin time, prothrombin time, thrombin time, fibrinogen, and antithrombin III), syphilis, and HIV tests.

A diffusion‐weighted brain MRI showed multiple bihemispheric hyperintense lesions in the territories of internal carotid arteries. CT angiography did not show significant stenosis of the anterior and posterior circulations. His TEE, 24‐hour Holter monitoring, and a transcranial Doppler showed no abnormalities. His chest CT showed lung cancer.

### 3.2 Case 2

A 79‐year‐old woman was admitted with confusion and mental dullness, which had begun 3 days before admission. Two months previously, she had had a bad temper and had become irritable; sometimes she had had hallucinations and delusions of persecution. She had undergone excision of a left eye cataract 5 months before admission.

On admission, she was confused and could not cooperate with the doctors. Laboratory tests, including blood routine test, serum electrolytes, renal and hepatic function, blood sugar, coagulation profile, syphilis and HIV tests, and blood fat, were all normal. There were no abnormalities on her ECG, TEE, 24‐hour Holter monitoring, or transcranial Doppler.

The DWI showed multiple high signal intensities in the territories of both internal carotid arteries and vertebrobasilar artery. Her chest CT scan showed lung cancer and multiple lung and bone metastases.

### 3.3 Case 3

A 68‐year‐old woman was admitted to the hospital with dizziness, disequilibrium, and dysarthria, which she had had for about 2 weeks. She had a history of hypertension, but no history of diabetes or heart disease, no familial history of cardiovascular disease, and no use of preventive medication for stroke.

On admission, her neurological examination was normal except for the presence of dysarthria, right central facial and glossary palsy, and bilateral positive Babinski signs.

Laboratory tests, including blood routine test, serum electrolytes, renal and hepatic function, blood sugar, coagulation profile, syphilis and HIV tests, and blood fat, were all normal. There were no abnormalities on her ECG, TEE, or 24‐hour Holter monitoring.

DWI showed multiple high signal intensities in the territories of both anterior and posterior circulations. MR angiography showed no stenosis of large vessels. Her chest CT scan showed lung cancer and multiple metastases in the lungs.

### 3.4 Case 4

A 60‐year‐old man was admitted with numbness and weakness of the left arm, which had begun 7 days before admission. His symptoms had remitted after about half an hour, but relapsed twice again. Except for the habit of smoking, he had no history of hypertension, diabetes or heart disease, and no familial history of cardiovascular diseases.

On admission, he was oriented and had no lymphadenopathy, no abnormal auscultation respiratory sound, and no palpable masses in his abdomen. His vital signs were normal. There was no abnormality on the ECG. His neurological examination was normal except for right mild central facial palsy and decreased pain in the right arm.

Laboratory tests, including blood routine test, serum electrolytes, renal and hepatic function, blood sugar, coagulation profile, syphilis and HIV tests, and blood fat, were all normal. There were no abnormalities on his TEE or 24‐hour Holter monitoring.

A diffusion‐weighted brain MRI showed multiple high signal intensities in the territories of both anterior and posterior circulations. CT angiography showed no culprit vessels. His chest CT scan showed lung cancer. The patient was diagnosed with squamous cell lung cancer by transbronchial lung biopsy.

### 3.5 Case 5

A 68‐year‐old man was admitted with dizziness and mental dullness, which he had had for 2 months; sometimes he had felt fatigue and had had intermittent fever. Bodyweight loss was about 5 kg. He had no history of hypertension, diabetes or heart disease, and no familial history of cardiovascular disease; but he did have habits of smoking and alcohol.

On admission, he was oriented and had no lymphadenopathy, no abnormal auscultation respiratory sound, and no palpable masses in his abdomen. His vital signs were normal. There was no abnormality on the ECG. His neurological examination was normal except for the dysfunction of cognition and bilateral positive Babinski signs.

Laboratory tests, including blood routine test, serum electrolytes, renal and hepatic function, blood sugar, coagulation profile, syphilis and HIV tests, and blood fat, were all normal, except for an erythrocyte sedimentation rate of 110 mm/h, carcinoembryonic antige of 153.2 ng/mL (normal, 0‐3.4 ng/mL), cancer antigen (CA)19‐9 of 89.63 U/mL (normal, 0‐39 U/mL), CA125 of 1615.00 U/mL (normal, 0‐35 U/mL), and albumin of 21.69 g/L (normal, 35.00‐55.00 U/mL). There were no abnormalities on his TEE or 24‐hour Holter monitoring.

DWI showed multiple high signal intensities in the territories of both anterior and posterior circulations. His chest CT scan showed lung cancer. Sputum smear detection showed adenocarcinoma tumor cells. CT angiography showed no stenosis of large vessels.

## 4 RESULTS

The mean age of our patients was 70 ± 7.31 years. The baseline clinical characteristics of these patients are shown in Table 1. All of the patients had lung cancers and had received no anti‐tumor treatment before stroke. Only one patient had no conventional common vascular risk factors of cerebral infarction. The common risk factors were hyperlipidemia, hypertension, and habits of alcohol and smoking. Three patients had increased D‐dimer levels, and one patient showed decreased antithrombin III levels. None of the patients had abnormal findings through TEE. 

**Table 1:** Basic clinical characteristics of the patients

	N	Sex	Age (years)	Symptom at onset	Cancer origin	Tumor pathology	Tumor stage	Vascular risk factors	Coagulation profile	D‐dimer level	TEE/TTE finding
This study	1	M	75	Dysarthria	Lung cancer	NA	NA	Hyperlipidemia	Normal	↑	Normal
	2	F	79	Psychiatric abnormal	Lung cancer	NA	NA	Alcohol	Normal	↑	Normal
	3	F	68	Dizziness and dysarthria	Lung cancer	NA	NA	Hypertension	Normal	Normal	Normal
	4	M	60	Hemiparesis, numbness	Lung cancer	Squamous cell	II	Smoking	Normal	↑	Normal
	5	M	68	Dizziness	Lung cancer	Adenocarcinoma	IV	No	AT III ↓61% (80%‐140%)	Normal	Normal
Park et al^12^	6	F	67	Dysarthria, hemiparesis	Ovarian carcinoma	NA	NA	No	Normal	Normal	Normal
	7	F	79	Dysarthria, hemiparesis	Pancreatic cancer	NA	IV	Hypertension, diabetes	APTT↑	↑	Normal
Kwon et al^3^	8	F	58	Unknown	Cholangioadenocarcinoma	Adenocarcinoma	Metastasis	NA	NA	↑	Normal
	9	M	67	Unknown	Pancreatic cancer	Adenocarcinoma	NA	NA	NA	↑	Normal
	10	M	62	Unknown	Lung cancer	Adenocarcinoma	Metastasis	NA	NA	NA	Normal
	11	F	73	Unknown	Acute myeloid leukemia	NA	NA	NA	NA	↑	Normal
	12	F	57	Unknown	Pancreatic cancer	NA	Metastasis	NA	NA	NA	Normal
	13	M	82	Unknown	Lung cancer	Squamous cell	Metastasis	NA	NA	NA	Normal
	14	F	70	Unknown	Unknown	Adenocarcinoma	Metastasis	NA	NA	↑	Normal
Bond et al^7^	15	F	36	Headache, impairment	Ovarian cancer	NA	NA	No	Normal	Normal	Normal
Chen et al^9^	16	M	65	Hemiplegia	Malignant monoclonal proliferative disease of B cell	B cell	NA	Hypertension	Normal	↑	Normal
	17	M	56	Hemiplegia, paresthesia	Lung cancer	NA	NA	No	APTT↑	↑	Normal
	18	M	50	Transient hemiplegia	Acute nonlymphocytic leukemia	NA	NA	No	FIB↓	↑	Normal
Costa et al^10^	19	F	74	Aphasia, crossed paralysis	Lung cancer	Non‐small cell	NA	Hypertension, dyslipidemia	FIB↓	↑	Normal
Kawasaki et al^11^	20	F	80	Unsteadiness of gait	Gastric cancer	Adenocarcinoma	NA	NA	Normal	↑	Normal
Tsai et al^13^	21	M	46	Diplopia, ophthalmoplegia	Colon cancer	Adenocarcinoma	NA	Diabetes, dyslipidemia	Normal	Normal	Normal

Abbreviations: APTT, activated partial thromboplastin time; AT III, antithrombin III; NA, not available; TEE/TTE, transesophageal echocardiography/transthoracic echocardiography.

Seven previous case series (16 patients) with DWI sequence were also analyzed in our study.^3^, ^7^, ^8^, ^9^, ^10^, ^11^, ^12^, ^13^ The mean age of the total patients (21 patients) was 66 years (mean ± SD, 66 ± 11.74 years). Lung cancer was the most common cancer with embolic stroke as the first manifestation. Pancreatic cancer was also common. Fifteen patients underwent D‐dimer level detection, and 11 of them showed highly increased levels. No positive result of these patients was found through TEE.

**FIGURE 1:**
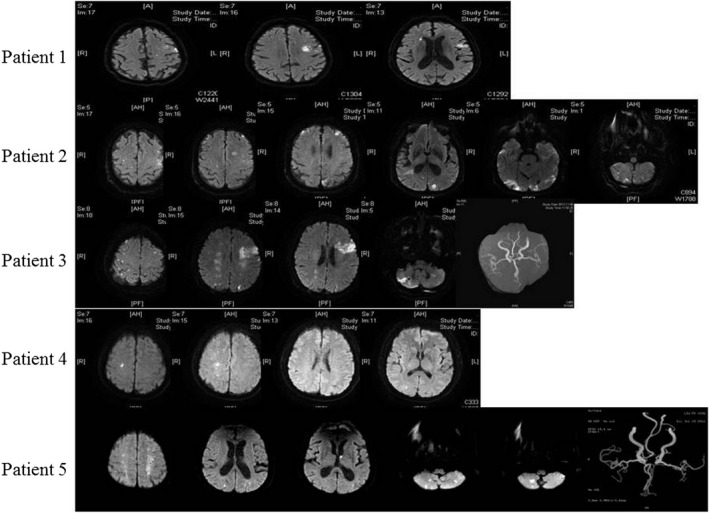
Diffusion‐weighted imaging patterns of our five patients.

The DWI features of our patients and those from the previous studies are shown in Table 2. Altogether, 22 (95.7%) patients had multiple and bilateral lesions. Eight (34.8%) patients had lesions in the supratentorium and 15 patients (65.2%) had lesions in both the supratentorium and infratentorium. Correspondingly, all of the patients had lesions involved in anterior circulations and 19 (82.6%) of them had lesions involved in both anterior and posterior circulations. Furthermore, the mechanisms of cerebral infarction from DWI features showed that all of the patients had embolic infarction, but eight (34.8%) of the patients had both embolic and watershed infarction. No culprit stenosis of the corresponding artery was found (Figure 1). MR angiographies, CT angiographies, or transcranial Doppler showed no culprit stenosis of the arteries in any of the patients.

**Table 2:** Diffusion‐weighted imaging features of the patients

Patient	Lesion number	Lesion distribution	Size	Site	Circulation involved	Infarction type
1	Multiple	Bilateral	Small	Supratentorium	Anterior	Embolic
2	Multiple	Bilateral	Small and large	Supratentorium and infratentorium	Anterior and posterior	Embolic
3	Multiple	Bilateral	Small and large	Supratentorium and infratentorium	Anterior and posterior	Embolic and watershed
4	Multiple	Bilateral	Small	Supratentorium	Anterior and posterior	Embolic and watershed
5	Multiple	Bilateral	Small	Supratentorium and infratentorium	Anterior and posterior	Embolic and watershed
6	Multiple	Bilateral	Small and large	Supratentorium and infratentorium	Anterior and posterior	Embolic and watershed
7	Multiple	Bilateral	Small and large	Supratentorium and infratentorium	Anterior and posterior	Embolic and watershed
8	Multiple	Bilateral	Small and large	Supratentorium and infratentorium	Anterior and posterior	Embolic and watershed
9	Multiple	Bilateral	Small and large	Supratentorium	Anterior and posterior	Embolic
10	Multiple	Bilateral	Small and large	Supratentorium	Anterior	Embolic
11	Multiple	Bilateral	Small and large	Supratentorium and infratentorium	Anterior and posterior	Embolic and watershed
12	Multiple	Bilateral	Small and large	Supratentorium and infratentorium	Anterior and posterior	Embolic
13	Multiple	Bilateral	Small and large	Supratentorium	Anterior and posterior	Embolic and watershed
14	Multiple	Bilateral	Small and large	Supratentorium and infratentorium	Anterior and posterior	Embolic
15	Multiple	Bilateral	Small	Supratentorium and infratentorium	Anterior and posterior	Embolic
16	Multiple	Bilateral	Small and large	Supratentorium and infratentorium	Anterior and posterior	Embolic
17	Multiple	Bilateral	Small	Supratentorium	Anterior and posterior	Embolic
18	Multiple	Left	Large	Supratentorium	Anterior	Embolic
19	Multiple	Bilateral	Small	Supratentorium and infratentorium	Anterior and posterior	Embolic
20	Multiple	Bilateral	Small	Supratentorium	Anterior	Embolic
21	Multiple	Bilateral	Small	Supratentorium and infratentorium	Anterior and posterior	Embolic

## 5 DISCUSSION

Cerebral infarction is not uncommon in patients with cancer. However, it is very rare that infarction presents as the first manifestation of a concealed cancer. Here, we present five patients with multiple cerebral infarctions as an initial presentation of occult cancer. Coincidentally, all of these five patients were diagnosed with lung cancer. This was inconsistent with other case series in which many different cancer origins showed infarction as their first manifestation. However, lung cancer was the most common origin with embolic brain stroke as the first manifestation. This is consistent with the study of Cestari et al.^14^ Pancreatic cancer was also a common type with embolic stroke as the initial presentation. The mean age of these patients was in their sixties and they had no common cause of embolic stroke.

The characteristics of stroke with cancer are usually multiple and embolic.^6^ DWI is a useful sensitive tool to identify small embolic infarcts. Early diagnosis of embolic stroke to identify occult cancer through DWI is very important and meaningful. The DWI of patients showed multiple small lesions involving bilateral anterior territories, and most of them had both anterior and posterior infarctions. All of the patients had supratentorium infarctions, and most of them had both supratentorium and infratentorium infarctions. Besides small embolic hyperintensities in DWI, watershed or borderzone infarction was also common in DWI. Compared with previous reports,^3^, ^12^ the case series also had such characteristics in DWI. That means multiple small bilateral infarctions, especially those involving both anterior and posterior circulations or supratentorium and infratentorium, are characteristics of stroke patients with occult cancer. Thus, patients with multiple small bilateral embolic infarctions, especially those involving both anterior and posterior circulations with unknown causes, should be examined for cancer or underlying malignancy.

Multiple small embolic infarctions are not unique characteristics of stroke patients with cancer. Cardiogenic, paradoxical, and aortogenic brain embolism are also common embolic sources. Differentiating the source of stroke is very important and difficult clinical work. According to the study by Fujimoto et al,^2^ aortogenic brain embolism was frequent in the vertebrobasilar system, but relatively rare in the right carotid system, while large infarctions were most common in cardiogenic brain embolism. Small infarctions or both small and large infarctions were more common in tumor emboli sources. Paradoxical brain embolism was more common in young patients and those with atrial septal aneurysm and patent foramen ovale. In the study by Jauss et al,^15^ patients with patent foramen ovale had a higher incidence of multiple lesions in the posterior circulation. Furthermore, TEE is useful in such patients. Thus, DWI patterns and TEE findings are very important for the differentiation of cancer embolism from cardiogenic, paradoxical, and aortogenic brain embolism.

Hypercoagulation is one of the mechanisms of cancer‐induced infarction. Tumor cells produce tumor necrosis factor or cytokines that promote coagulation and form microscopic thrombi by inducing disseminated intravascular coagulation. In addition, nonbacterial thrombotic endocarditis induced by aggregation and adhesion of fibrin and platelets to the cardiac valves can also cause cerebral infarction.^16^ Tumor embolism, most commonly with primary or metastatic neoplasms of the lung, is a rare cause of cerebral infarction. Infarcts from tumor emboli are typically larger than those seen with nonbacterial thrombotic endocarditis. Non‐bacterial thrombotic endocarditis is the most common etiology for stroke in cancer patients.^14^ However, it is difficult to diagnose through TEE. Highly increased D‐dimer level may indicate hypercoagulation and may be associated with cancer‐related stroke.^17^ However, abnormal findings could be seen in the coagulation profiles of our patients and previous case series. This may also indicate the dysfunction of the coagulation system. Therefore, the mechanisms of embolic infarctions associated with cancers in our patients could not be confirmed.

Although this study was conducted in a small number of cases, it may indicate that brain multiple embolisms, especially small and bilateral infarctions involved in both anterior and posterior circulations in DWI with unknown causes, could be the first manifestation of occult cancer. Early diagnosis of occult cancer is beneficial for initiating the early management of cancer patients.

## CONFLICTS OF INTEREST

Xiaosa Chi and Xueping Zheng: Writing of paper. Yongjun Mao and Xueping Zheng: Design, literature review, coordination. Xiaosa Chi, Renliang Zhao and Haitao Pei: Literature review, data collection. Ang Xing and Song Hu : Data collection, review of medical records, initial statistical analysis. Xiaosa Chi and Jingjiao Chen: Data collection, second statistical analysis.
